# Increased vulnerability to SARS-CoV-2 infection among indigenous people living in the urban area of Manaus

**DOI:** 10.1038/s41598-021-96843-1

**Published:** 2021-09-02

**Authors:** Gemilson Soares Pontes, Jean de Melo Silva, Renato Pinheiro-Silva, Anderson Nogueira Barbosa, Luciano Cardenes Santos, Antônio de Pádua Quirino Ramalho, Carlos Eduardo de Castro Alves, Danielle Furtado da Silva, Leonardo Calheiros de Oliveira, Allyson Guimarães da Costa, Ana Carla Bruno

**Affiliations:** 1 Laboratório de Imunologia e Virologia, Instituto Nacional de Pesquisas da Amazônia (INPA), Coordenação Sociedade, Ambiente e Saúde, Av. André Araújo, 2936-Petrópolis, Manaus, Amazonas CEP 69.067-375 Brazil; 2 Universidade Federal do Amazonas (UFAM), Programa de Pós-Graduação em Imunologia Básica e Aplicada, Manaus, Brazil; 3 Universidade do Estado do Amazonas, Programa de Pós-Graduação em Ciências Aplicadas à Hematologia, Manaus, Brazil; 4 Universidade Federal do Mato Grosso, Campus do Araguaia, Barra das Garças, Mato Grosso Brazil; 5 Departamento de Saúde Coletiva, Universidade Federal do Amazonas (UFAM), Faculdade de Medicina, Manaus, Brazil; 6 Instituto Nacional de Pesquisas da Amazônia (INPA), Programa de Pós-Graduação em Biodiversidade e Biotecnologia da Amazônia Legal-PPG-BIONORTE, Manaus, Brazil; 7 Escola de Enfermagem de Manaus, Universidade Federal do Amazonas (UFAM), Manaus, Brazil

**Keywords:** Infectious diseases, Viral infection, Epidemiology

## Abstract

The COVID-19 pandemic threatens indigenous peoples living in suburban areas of large Brazilian cities and has thus far intensified their pre-existing socio-economic inequalities. We evaluated the epidemiological situation of SARS-CoV-2 infection among residents of the biggest urban multiethnic indigenous community of the Amazonas state, Brazil. Blood samples of 280 indigenous people living in the surrounding area of Manaus were tested for the presence of anti-SARS-CoV-2 IgA or IgG antibodies. The risk factors and sociodemographic information were assessed through an epidemiological questionnaire. We found a total positivity rate of 64.64% (95% CI 59.01–70.28) for SARS-CoV-2 infection. IgA and IgG were detected in 55.71% (95% CI 49.89–61.54) and 60.71% (95% CI 54.98–66.45) of the individuals, respectively. Over 80% of positive individuals were positive for both IgA and IgG.No significant difference in positivity rates between genders or age groups was observed. Moreover, the age group ≥ 60 years old showed the highest antibody ratios (IgA mean ratio = 3.080 ± 1.623; IgG mean ratio = 4.221 ± 1.832), while the age groups 13–19 and 20–29 showed the lowest IgA (mean ratio = 2.268 ± 0.919) and IgG ratios (mean ratio = 2.207 ± 1.246), respectively. Individuals leaving the home more frequently were at higher risk of infection (Odds ratio (OD) 2.61; 95% CI 1.00–1.49; p = 0.048). Five or more individuals per household increased fivefold the risk of virus transmission (OR 2.56; 95% CI 1.09–6.01; p = 0.019). The disproportionate dissemination of SARS-CoV-2 infection observed among the study population might be driven by typical cultural behavior and socioeconomic inequalities. Despite the pandemic threat, this population is not being targeted by public policies and appears to be chronically invisible to the Brazilian authorities.

## Introduction

The state of Amazonas in northern Brazil is one of the states that has been hit hardest by the current pandemic. In the period between the WHO declaring the COVID-19 pandemic on March 11, 2020 and the end of March 2021^1^, the death toll from COVID-19 in the Amazonas state has surpassed the twelve thousand mark. The Amazonas state is located in a region of paramount environmental and demographic importance. The state comprises one-fifth of the Brazilian territory and contains approximately 40% of the world’s rainforests^2^. The majority of the population in the state has an indigenous mixed-heritage background, and sixty percent of its 4 four million inhabitants live in the capital Manaus^3^.

Based on the last population census, around 168,000 indigenous peoples are distributed in 641 communities and 62 ethnic groups live within Amazonas state^4^. Indigenous populations have to deal with many social inequalities resulting from socio-economic marginalization, including poor nutrition, lack of access to health services and proper sanitation^5^. This situation put indigenous communities under worrying threat during public health emergencies, such as the coronavirus pandemic.

The number of indigenous people affected by COVID-19 in Brazil is not accurately notified^6^. According to the Special Secretariat of Indigenous Health (SESAI), over 600 individuals died from COVID-19 in Brazil and the state of Amazonas is responsible for 25% of this number^7^. However, the death rates must be much higher than the official numbers. Also, the epidemiological data is not stratified by ethnicity, which could allow identifying related risk factors^8^. On top of this, Brazil’s indigenous agencies do not monitor the COVID-19 epidemiological situation in urban indigenous communities^9^.

There are approximately 315,000 indigenous people grouped in 300 ethnicities living close to the large cities of Brazil^4^. However, the true number of indigenous populations residing in the surrounding areas of Manaus is unknown, though, it is estimated that 20,000 individuals from 92 ethnicities live in 62 urban indigenous communities^10^. This population is completely invisible to the Brazilian public health agenda, especially regarding COVID-19. Thus, this study assessed the magnitude of the spread of SARS-CoV-2 and its transmission risk factors among the biggest multiethnic urban indigenous community located on the west side of Manaus, Amazonas state, Brazil.

## Material and methods

### Ethical approval

This study was approved by the Brazilian Commission for Ethics in Research-CONEP (approval number: 4.260.763). All individuals signed the informed consent form before taking part in this study. An informed consent was obtained from a parent and/or legal guardian of the participants under the age of 18 years old. The confidentiality and the right to leave the study at any time were guaranteed to all participants. All experiments were performed in accordance with relevant guidelines and regulations.

### Study design and population

Between October 10 and November 14, 2020, we randomly recruited indigenous residents in the community of Parque das Tribos, the biggest multiethnic urban indigenous community in Manaus. Over 1300 indigenous people from 35 different ethnicities live in this community, which is located in the Tarumã district, west Manaus, Amazonas state, Brazil. This study applied consecutive sampling as a selection strategy and the sample size was statistically estimated using a 95% confidence interval.

**Figure 1:**
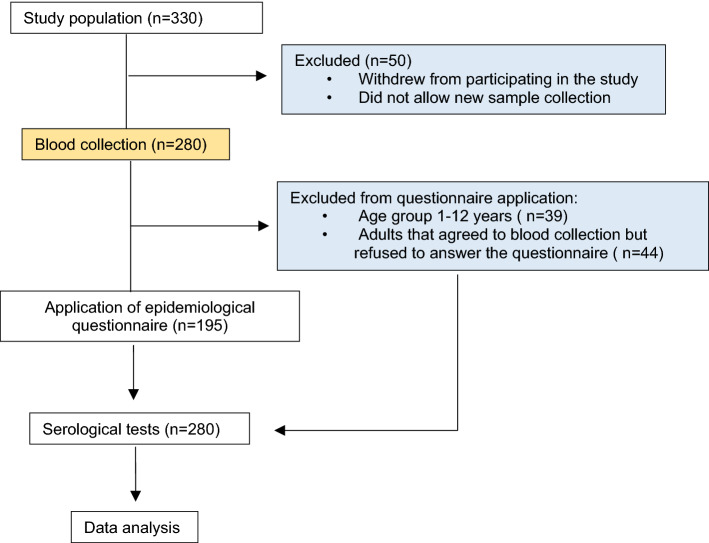
Flow chart shows study design and the number of individuals included.

From a total of 280 individuals of both genders aged from 1 to 83 years, and from different ethnicities, approximately 5 ml of peripheral blood was collected by venipuncture for posterior serological analysis. A standardized interviewer-administered questionnaire was used to obtain information on sociodemographic and risk factor variables. Because of the questionnaire’s complexity, it was decided by a multidisciplinary team (composed of anthropologists, nurses, physicians, virologists and epidemiologists) that only individuals over 13 years old would answer the questionnaire in order to guarantee the quality of the results obtained in the survey^11^. Some adults (n = 43) agreed to have blood collected, but refused to respond the questionnaire due to cultural issues or due to feeling uncomfortable doing so (Fig. 1).

The COVID-19-related symptoms were assessed through a questionnaire applied by a physician and nurses from the project team. The symptoms were classified as mild and moderate according to the WHO guidelines^12,13^. Those individuals with COVID-19-related symptoms during the blood collection or seropositive for SARS-CoV-2 were instructed by a physician according to the protocols and referral pathway established by the WHO guidance^12,14^.

### Serological analysis

Serum samples from the study population were tested for SARS-CoV-2 IgA and IgG antibodies (Abs) through an enzyme-linked immunosorbent assay (*Anti-SARS-CoV-2 IgA/IgG ELISA-euroimmun, Germany)*, which was performed according to the manufacturer's instructions^15^. The optical density was measured in a spectrophotometer (Epoch Microplate, Biotek Instruments Inc. USA) using a 450 nm filter, and the test positivity was determined according to the cut-off formula indicated by the manufacturer. Cut-off ranges were obtained by calculating a ratio of the optical density (OD) values of the control or patient sample over the OD of the calibrator, according to the following formula: OD of the control or patient sample/OD of calibrator = ratio. Ratios < 0.8 were considered negative and ratios ≥ 1.1 positive. Ratios 0.8≥ to < 1.1 were considered borderline. Individuals with borderline results were retested twice within 2–4 weeks. According to the manufacturer, the specificity and sensibility for anti-S IgG ≥ 21 days post-symptom onset are 100% and 99.3%, respectively. For anti-S IgA, the specificity is 100% and sensibility is 92.4%, ≥ 10 days post post-symptom onset. Serological tests were performed at the Laboratory of Virology and Immunology of the National Institute of Amazonian Research (INPA).

### Statistical analysis

Descriptive statistical analysis was used to evaluate the sociodemographic variables and serum levels of SARS-Cov-2 antibodies observed in the study population. The prevalence rates were estimated based on the positivity for anti-SARS-CoV-2 IgG, while the total positive rates were estimated based on the positivity for anti-SARS-CoV-2 IgG or IgA. Multivariate analysis using logistic regression was performed to assess the relationship between sociodemographic characteristics and IgG positivity to SARS-CoV-2 infection. The odds ratio (OR) analysis was performed on the following variables: genders, age, marital status, level of schooling, housing type, COVID-19 information sources and frequency of leaving the home. One-way ANOVA and a Tukey post hoc test were used to compare serum levels of SARS-CoV-2 antibodies (optical density values) among age ranges. The effect of household crowding on the risk of exposure to infection was evaluated through OR analysis and Fisher’s exact test. The statistical analyses were performed using the R v.4.0.4 and the Toolkit v.3.0.1 software. In all analyses, a value of p < 0.05 was considered significant. A flowchart showing the steps taken in the study is shown below (Fig. 1).

### Consent to participate

All the patients included in this study signed a written consent form.

### Consent for publication

All authors have read and agreed to the published version of the manuscript.

## Results

### Positivity rates of SARS-CoV-2 infection

**Figure 2:**
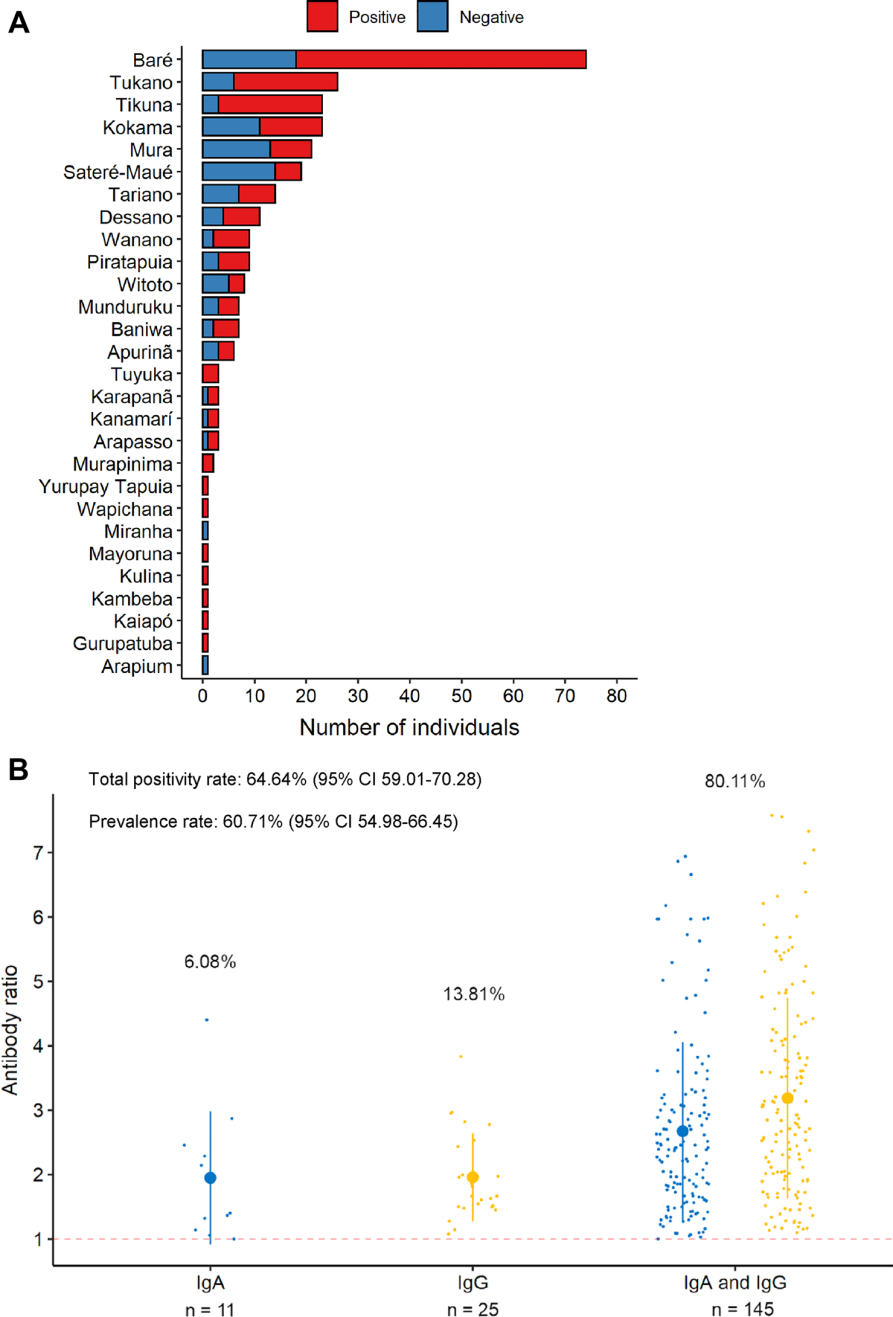
Ethnic diversity of the study population and positivity rates. (**A**) SARS-CoV-2 positivity according to ethnicity. (**B**) Pattern of IgA, IgG and IgA/IgG positivity among infected individuals. Smaller dots represent the antibody ratio from each individual. Larger dots and vertical lines represent mean and standard deviation, respectively. Dashed line represent cut-off values specified by the manufacturer.

The study population was composed of 28 different indigenous ethnic groups. Baré, Tukano, Tikuna and Kokama were the most common ethnicities (Fig. 2A). A total of 181 individuals (64.64%; 95% CI 59.01–70.28) were positive for SARS-CoV-2 specific IgA or IgG antibodies (Abs). The positivity rates for IgA and IgG observed in the study population were 60.71% (95% CI 54.98–66.45) and 55.71% (95% CI 49.89–61.54), respectively. From the total number of individuals positive for SARS-CoV-2, 145 (80.11%; 95% CI 74.24–85.98) tested positive for both IgA and IgG, whereas 11 (6.07%; 95% CI 2.56–9.59) and 25 (13.81%; 95% CI 8.72–18.85) individuals were positive only for IgA or IgG, respectively (Fig. 2B).

**Figure 3:**
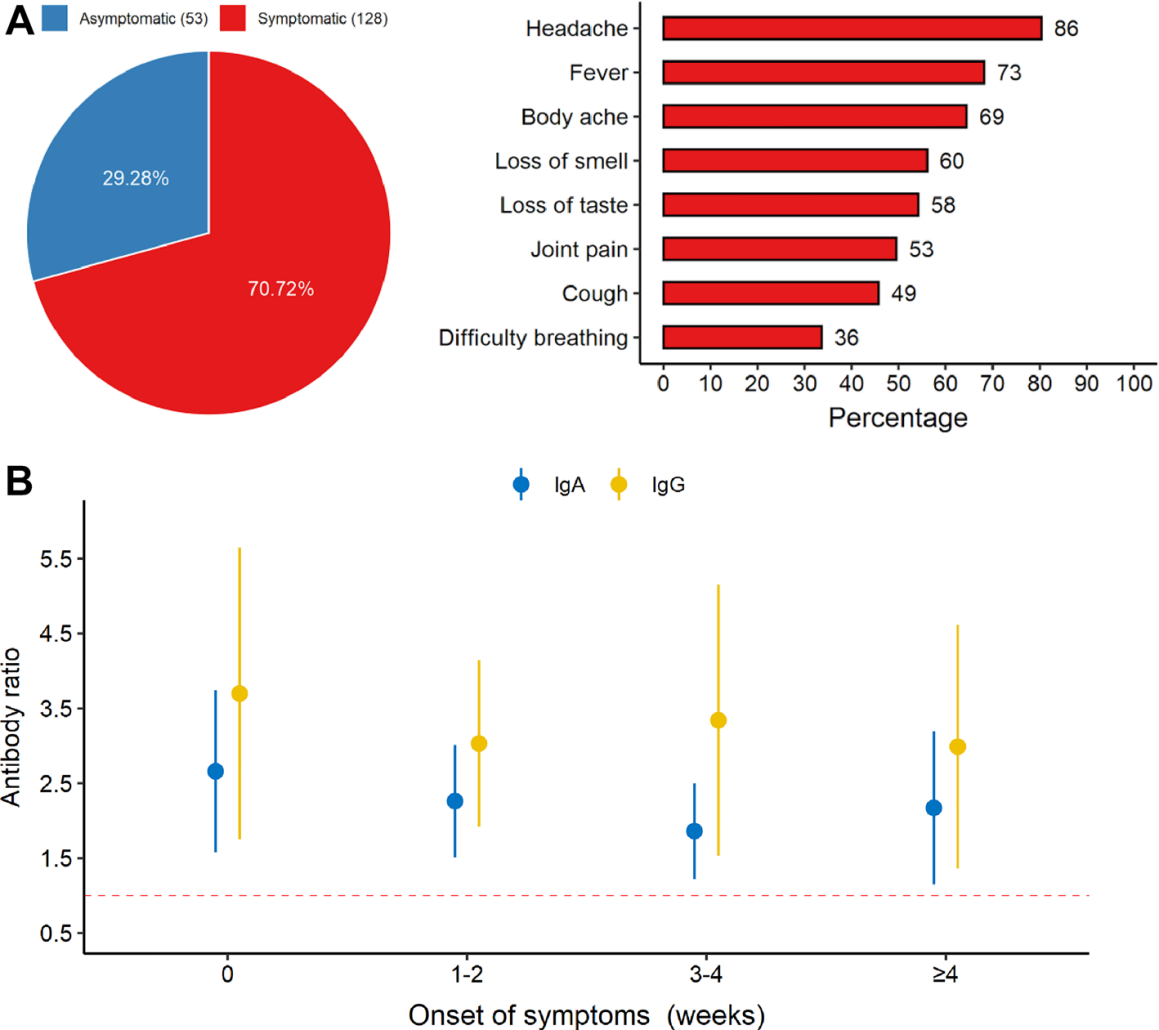
Main clinical symptoms and pattern of positivity according to the day of onset of the individual’s symptoms. (**A**) Number of symptomatic and asymptomatic individuals positive for SARS-CoV-2. (**B**) IgA and IgG positivity rates according to the day of the onset of the symptoms. Number 0 means the individuals presented symptoms on the day of blood collection. Dots and vertical lines represent mean and standard deviation, respectively. Dashed line represent cut-off values specified by the manufacturer.

Mild or moderate COVID-19-related symptoms were present in 107 individuals (59.11%) who were positive for SARS-CoV-2. The most common symptoms were headache, fever, body aches, loss of taste or smell and/or joint pain (Fig. 3A). All individuals with symptoms on the day of blood collection (n = 11) were positive for both IgA and IgG, while 88.71% (n = 55) of the individuals were still displaying high serum levels of IgA even 4 weeks after the onset of their symptoms (Fig. 3B).

### Serological profile according to the social demographical characteristics

The total positivity rates were similar between women and men (p = 0.207) and no significant differences between age groups were observed (Table 1). However, when the IgG positivity rates were compared based on antibody ratios, the difference observed between the following age groups was statistically significant: ages 20–29 and 40–49 (p = 0.044); 13–19 and ≥ 60 (p = 0.002); 20–29 and ≥ 60 (p = 0.0004) (Supplementary 1A). People belonging to the age group ≥ 60 years old showed the highest antibody ratios (IgA mean ratio = 3.080 ± 1.623; IgG mean ratio = 4.221 ± 1.832), while the age groups 13–19 and 20–29 showed the lowest IgA (mean ratio = 2.268 ± 0.919) and IgG ratios (mean ratio = 2.207 ± 1.246), respectively. These values may indicate the serum level of antibodies in these individuals, however, in order to confirm this information it is necessary to perform antibody titration. Due to budget limitations, we were not able to assess the antibody serum levels.

**Table 1:** SARS-CoV-2 positivity according to sociodemographic characteristics of the study population.

Sociodemographic characteristics	N (%)	Age range	IgA N (%)	IgG N (%)	IgA and IgG N (%)	IgA or IgG N (%)	OR (95% CI)	OR p-value
**Gender**								
Female	162 (57.9)	03–83	83 (51.2)	95 (58.6)	77 (47.5)	101 (62.3)	Ref.	
Male	118 (42.1)	01–79	73 (61.9)	75 (63.6)	68 (57.6)	80 (67.8)	1.27 (0.77–2.10)	0.347
**Age**								
1–12	39 (13.9)		20 (51.3)	24 (61.5)	20 (51.3)	24 (61.5)	Ref.	
13–19	36 (12.9)		18 (50.0)	23 (63.9)	16 (44.4)	25 (69.4)	1.42 (0.54–3.70)	0.473
20–29	39 (13.9)		23 (59.0)	24 (61.5)	20 (51.3)	27 (69.2)	1.41 (0.55–3.59)	0.476
30–39	48 (17.1)		18 (37.5)	25 (52.1)	18 (37.5)	25 (52.1)	0.68 (0.29–1.60)	0.377
40–49	48 (17.1)		35 (72.9)	33 (68.8)	33 (68.8)	35 (72.9)	1.68 (0.68–4.16)	0.260
50–59	36 (12.9)		23 (63.9)	24 (66.7)	21 (58.3)	26 (72.2)	1.63 (0.61–4.30)	0.328
≥ 60	34 (12.2)		19 (55.9)	17 (50.0)	17 (50.0)	19 (55.9)	0.79 (0.31–2.02)	0.624
**Marital status**								
Single	89 (45.6)	14–71	51 (57.3)	54 (60.7)	44 (49.4)	61 (68.5)	Ref.	
Married or living together	101 (51.8)	18–83	55 (54.5)	59 (58.4)	52 (51.5)	62 (61.4)	0.73 (0.40–1.33)	0.304
Widower	5 (2.6)	43–71	3 (60.0)	2 (40.0)	2 (40.0)	3 (60.0)	0.69 (0.11–4.35)	0.692
**Level of schooling**								
Up to middle school	46 (23.6)	13–83	22 (47.8)	23 (50.0)	19 (41.3)	26 (56.5)	Ref.	
Middle school completed	19 (9.7)	15–73	11 (57.9)	11 (57.9)	10 (52.6)	12 (63.2)	1.32 (0.44–3.96)	0.622
High school incomplete	45 (23.1)	14–64	25 (55.6)	26 (57.8)	22 (48.9)	29 (64.4)	1.39 (0.60–3.24)	0.440
High school completed	58 (29.7)	17–68	33 (56.9)	35 (60.3)	29 (50.0)	39 (67.2)	1.58 (0.71–3.52)	0.263
In higher education	27 (13.9)	18–79	19 (70.4)	21 (77.8)	19 (70.4)	21 (77.8)	2.69 (0.92–7.92)	0.072
**COVID-19 information sources**								
TV	63 (32.3)	14–83	31 (49.2)	35 (55.6)	29 (46.0)	37 (58.7)	Ref.	
Internet	20 (10.3)	13–56	11 (55.0)	11 (55.0)	9 (45.0)	13 (65.0)	1.31 (0.46–3.72)	0.618
TV and Internet	34 (17.4)	16–79	16 (47.1)	18 (52.9)	14 (41.2)	20 (58.8)	1.00 (0.43–2.34)	0.993
Others ( religious leader or friends)	78 (40.0)	13–75	52 (66.7)	52 (66.7)	47 (60.3)	57 (73.1)	1.91 (0.94–3.87)	0.074
**Frequency of leaving the home**								
Rarely	34 (17.4)	14–74	16 (47.1)	17 (50.0)	15 (44.1)	18 (52.9)	Ref.	
Once a week	102 (52.3)	13–83	57 (55.9)	59 (57.8)	51 (50.0)	65 (63.7)	1.56 (0.71–3.42)	0.266
2–4 times a week	59 (30.3)	15–64	37 (62.7)	40 (67.8)	33 (55.9)	44 (74.6)	2.61 (1.07–6.37)	0.035*

*OR* Odds Ratio obtained through multivariate analysis using logistic regression, *CI* Confidence Interval.

p < 0.05 (*).

The level of education of the study population was considered low. Only 29.7% of the study population had received a high school diploma (Table 1). The level of social isolation was directly related to the increased risk of infection. People who declared leaving the home 2–4 times a week were more susceptible to infection (95% CI 1.00–1.49; p = 0.044) (Table 1). The main reasons for leaving the home were either to work or buy food.

**Figure 4:**
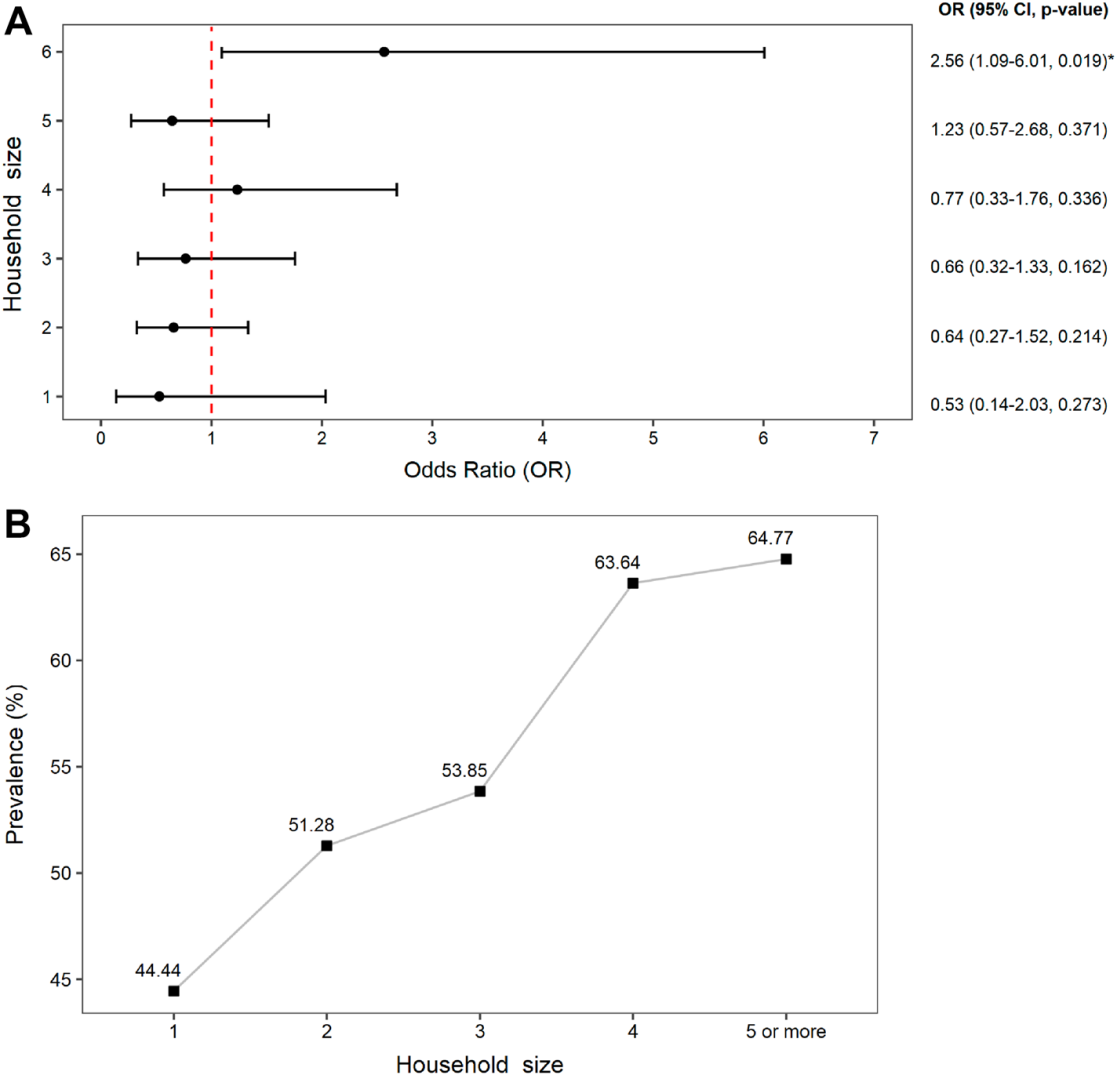
Prevalence rates by household size. (**A**) Association between the risk of exposure to infection and the household size (number of members). (**B**) SARS-CoV-2 prevalence rates according to the number of inviduals per resindence. OR Fisher’s exact test (*p < 0.05).

As a typical Brazilian indigenous house is small and overcrowded, since usually it serves as a collective house (two or more families), we evaluated the influence of dwelling size on the prevalence of SARS-CoV-2 infection. Our findings demonstrate that the higher the number of individuals per house the higher the susceptibility to SARS-CoV-2 infection. The susceptibility of people living with five people or more is almost fourfold higher when compared with people living with two people and almost fivefold higher when compared with people living with one other person (95% CI 1.96–6.01; p = 0.010) (Fig. 4A). The prevalence observed also progressively increased as the number of individuals per home rose (Fig. 4B). The highest prevalence (64.77%) was observed among individuals living with five or more people.

**Figure 5:**
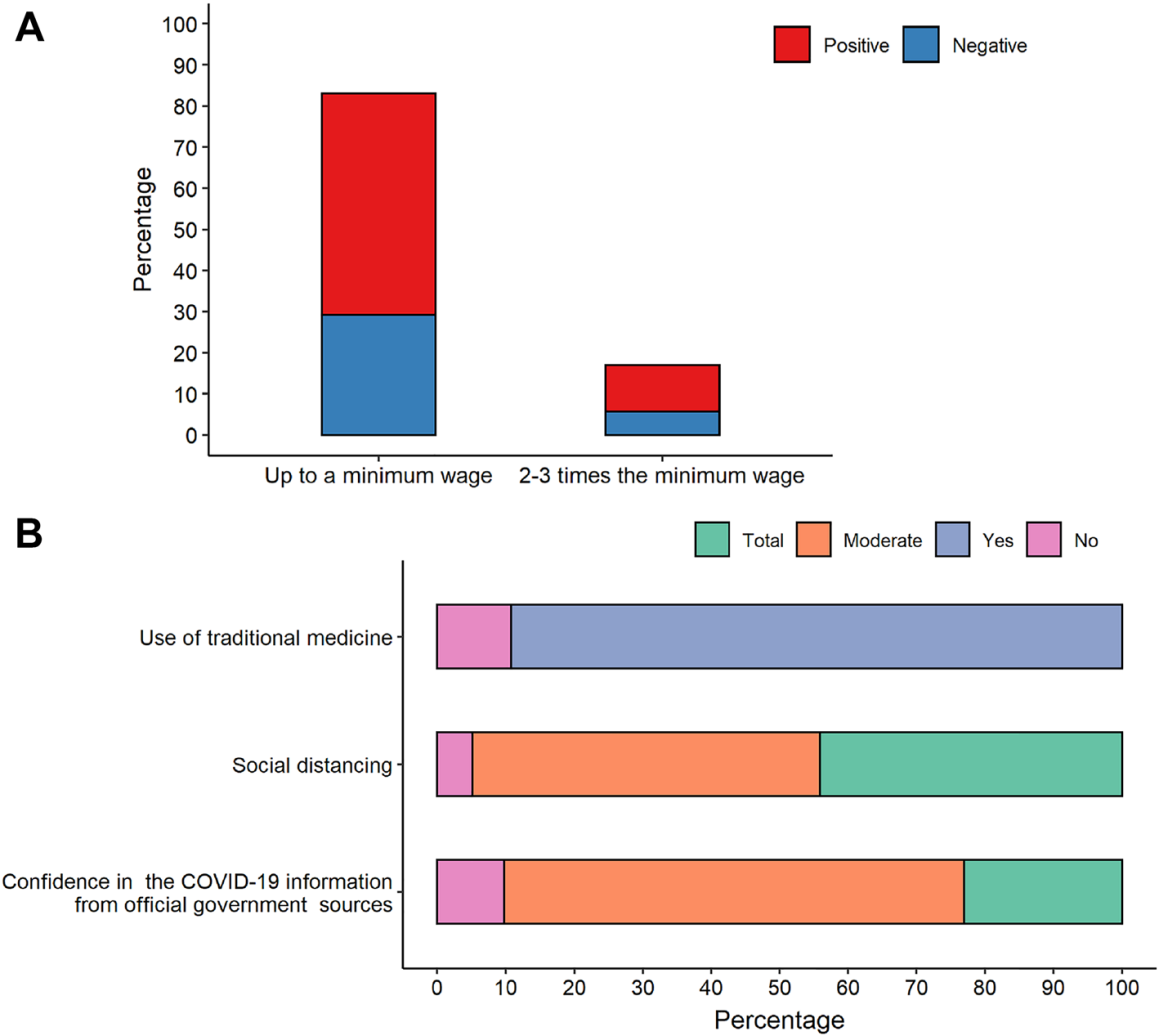
(**A**) Purchasing power and behavior characteristics (**B**) of study population.

The level of purchasing power of the study population was very low. Over 83% of individuals earn up to one minimum wage (approximately USD 192 dollars in Brazil), which hampers effective access to adequate nutrition and healthcare assistance (Fig. 5A). Additionally, the majority of the study population (90%) declared using traditional medicine to treat or prevent COVID-19 and other infectious diseases. Most of the individuals (> 60%) declared that they partially trust the official Brazilian government COVID-19-related information. According to their answers, only 5.13% did not practice social distancing (Fig. 5B).

## Discussion

The Brazilian northern region poses the highest rate of SARS-CoV-2 infection vulnerability. By late March 2021, Amazonas state concentrated the most deaths per million people^16^. As such, the effectiveness of pandemic response management demands a better comprehension on the impact of social asymmetries in relation to SARS-CoV-2 exposure risk within different population groups, especially amid ethnic minorities living in poverty, such as the indigenous populations of this region.

To our knowledge, this is the first report on the epidemiological situation of SARS-CoV-2 infection in a Brazilian indigenous urban community. Our findings demonstrate that SARS-CoV-2 is highly disseminated among the study population since it showed a prevalence rate of 60.71%. Similar prevalence was observed in the rural indigenous village of Xikrin of Bacajá (Kayapó), in the Brazilian state of Pará, which is also in the northern region^17^. Moreover, a population-based study conducted in Manaus during the same period of our study identified a prevalence of 29.10% while the Brazilian National SARS-CoV-2 prevalence estimated in June of 2020 was 3.1%^18,19^. These data clearly show that indigenous people are disproportionately hit by SARS-CoV-2 infection. This scenario might be a result of social vulnerabilities, which in turn maximize dissemination of the virus and put this population at greater risk.

SARS-CoV-2 strikes ethnic minorities who experience multidimensional poverty much harder, principally the indigenous peoples that constitute about 15% of the population living in extreme poverty in Brazil^20^. This population struggles to adopt measures to prevent and mitigate the SARS-CoV-2 spread since they have limited access to basic services like adequate sanitation and clean water. The study population was composed of families of low education and income levels. Our findings showed that over 95% of the residents of Parque das Tribos had no access to clean drinking water and adequate sanitation. The precarious and chaotic social conditions of the study population could be the main reason for the high positivity rates observed.

The positivity rate for anti-SARS-CoV-2 IgA Abs found in this study was 55%. IgA is a dimeric polyvalent antibody found predominantly in mucosal surfaces of humans and other mammals^21^. The role of this antibody in the course of SARS-CoV-2 infection is not completely understood, but it might be crucial to the pathogenesis of COVID-19. IgA seems to more effectively neutralize the SARS-CoV-2 than other antibody subclasses^22,23^. Furthermore, serum IgA appears earlier than IgM and IgG in SARs-CoV-2 infected individuals and may persist for several weeks after complete recovery^24^. In the present study, a high IgA ratio was detectable in almost 90% of individuals after more than four weeks of the onset of their symptoms. All individuals with symptoms on the day of blood collection were positive for both IgA and IgG. It is possible that the seroconversion for IgA and IgG happened simultaneously in these individuals, as previously reported^25^. From the total number of infected individuals, 80.11% were positive for both IgA and IgG. As IgA has a high potential for virus neutralization and may last for a long time, the course of SARS-CoV-2 infection could be influenced by its serum levels^22^. However, we did not follow up the study population to observe the clinical outcome of the infection. More comprehensive studies are necessary in order to confirm this speculation.

The anti-SARS-CoV-2 IgA and IgG ratios gradually rose with age and elderly individuals (≥ 60 years old) showed the highest antibody ratios. However, no significant difference in positivity rates was observed between the age groups. A nationwide study conducted with non-indigenous people in the United States showed that working adults (20–49 years old) were at a higher transmission risk^26^. Also, a study demonstrated that non-indigenous Brazilian people aged 30–39 and 40–49 years old were at the highest risk of SARS-CoV-2 infection^19^. The authors suggested that these age groups might be more susceptible or exposed to SARS-CoV-2 infection because they are working adults who must leave home to support their families. Indeed, we observed that the individuals of the study population who left home more often (2–4 times a week) had an increased infection risk. The main reasons for leaving home were either to work or to buy food.

Housing is also an important factor that affects SARS-CoV-2 dissemination among indigenous people. Keeping your distance from others in an indigenous home is not often feasible because they are often overcrowded and very small, which enhances the transmission of the virus^16^. Our findings demonstrated that individuals living in households with six or more people had an almost fivefold increase in risk. This situation is worsened by poor-quality housing. The dwellings of Parque das tribos are very small buildings constructed with low-quality materials, without essential basic infrastructure such as a supply of safe drinking water or effective sewerage. Thus, housing conditions are completely inadequate for keeping them safe from many communicable diseases, including COVID-19.

The majority of the study population declared to adopt distancing measures during the pandemic. However, this might be a huge challenge to these individuals due to their socioeconomic liabilities along with the difficult access to COVID-19-related information^27^. Although access to information is a human right, providing accurate information about COVID-19 to indigenous peoples is very complicated. They face difficulties due to language barriers and in accessing different platforms to get updated information. As a consequence, many individuals do not trust the official government information and consult religious leaders for guidance related to different aspects of their lives, including health issues^28^. Our data also showed that most of the individuals partially trust the information coming from the Brazilian government.

Furthermore, about 90% of the study population reported using medicinal plants or herbs to treat or prevent SARS-CoV infection. The use of medicinal herbs is an important part of indigenous culture and must be respected. Traditional knowledge is the foundation of the available conventional treatments and is the cornerstone of pharmacological research^29,30^. However, the use of herbs can cause a false sensation of protection, which may result in the discontinuation of important measures to prevent COVID-19 and enhance the vulnerability of these populations.

Despite the limitation of our data, this study indicates that indigenous peoples living in urban areas are being dramatically affected by SARS-CoV-2, especially because of their poor socioeconomic conditions and lack of access to adequate health assistance. This population is fighting a double battle due to the fact that a) in the Brazilian national health system (SUS), their indigenous identity is not recognized by the patient management system and b) They are not assisted by SESAI because they are outside their villages or reservations. Both situations reinforce the invisibility of these populations. Thus, we need coordinated national actions that prioritize ethnic vulnerable groups in the battle against COVID-19. We need public policies that promote health, adequate housing and sanitation for these populations. Otherwise, indigenous people living in urban areas are doomed to suffer at unprecedented levels during the current pandemic.

## Supplementary Information

Supplementary Information.

## Data Availability

All data generated or analyzed during this study are included in this published article and its supplementary information files.
