# Supplementary figures and images for: Increased vulnerability to SARS-CoV-2 infection among indigenous people living in the urban area of Manaus
Source: Sci Rep. 2021 Sep 2;11:17534. doi: 10.1038/s41598-021-96843-1 (PMC8413354; doi:10.1038/s41598-021-96843-1)

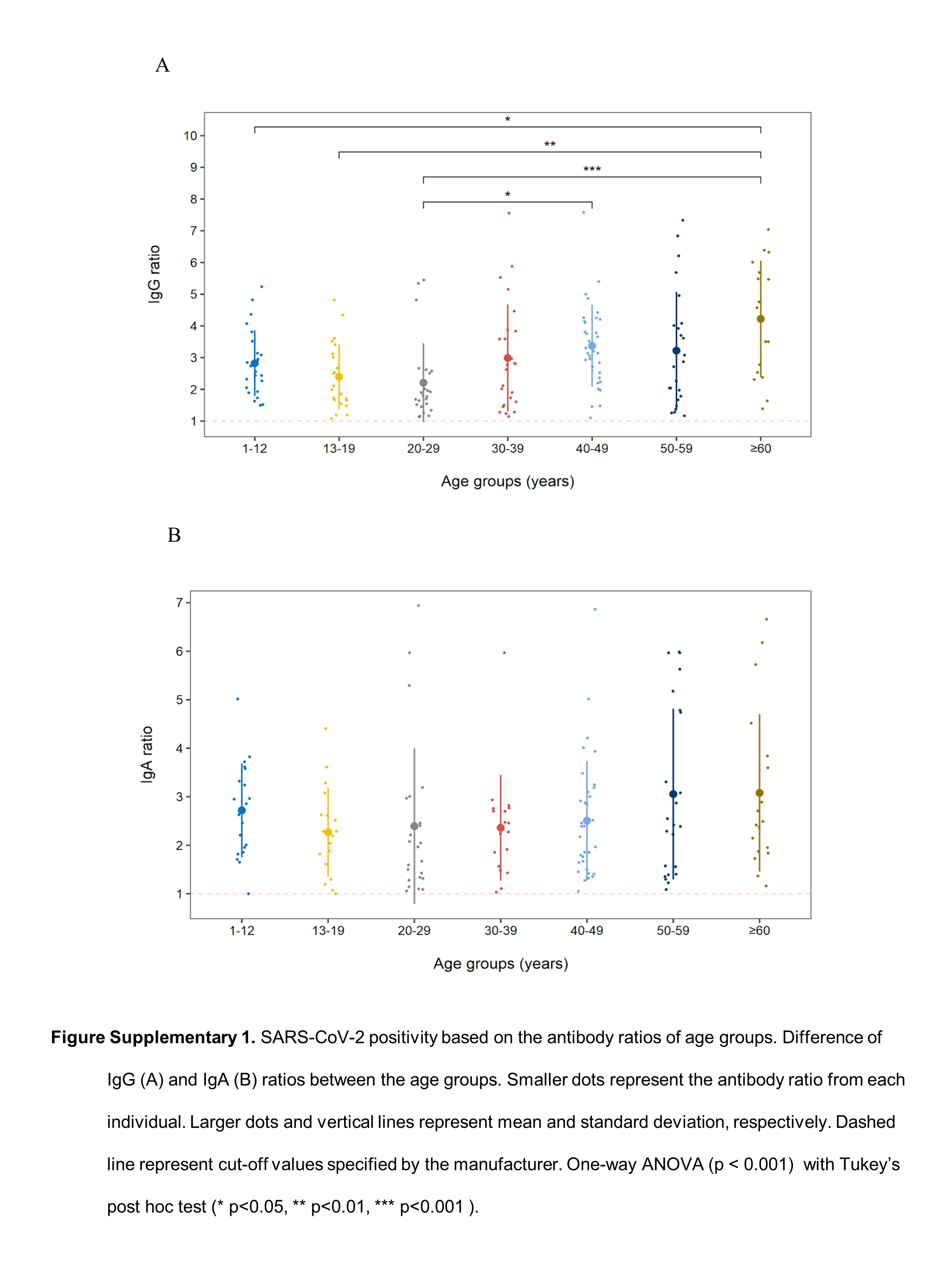

Supplement: Supplementary file 1 — Supplementary Information. [file 41598_2021_96843_MOESM1_ESM.tif]
